# Pilot Validation of a Novel Inline Device for Real-Time Monitoring of Abdominal Mechanics During Pneumoperitoneum

**DOI:** 10.3390/ani16111593

**Published:** 2026-05-23

**Authors:** Marta Guadalupi, Roberta Belvito, Floriana Cavalluzzo, Pietro Francesco Pio Magli, Agata Fraccascia, Francesco Staffieri, Luca Lacitignola

**Affiliations:** 1 Dipartimento DiMePRe-J, Sez. Cliniche Veterinarie e p.a., Campus di Medicina Veterinaria, Università Degli Studi di Bari “Aldo Moro”, s.p. per Casamassima km 3, Valenzano, 70010 Bari, Italy; marta.guadalupi@uniba.it (M.G.); r.belvito@phd.uniba.it (R.B.); f.cavalluzzo@phd.uniba.it (F.C.); agata.fraccascia@uniba.it (A.F.); francesco.staffieri@uniba.it (F.S.); 2 PhD Course SCICOV, Campus di Medicina Veterinaria, University of Bari, 70010 Bari, Italy; p.magli1@phd.uniba.it

**Keywords:** abdominal compliance, pressure–volume curve, laparoscopy, pneumoperitoneum, veterinary surgery, biomechanical monitoring, insufflation, inline device

## Abstract

The abdominal pressure–volume (P–V) relationship during laparoscopic insufflation is curvilinear and subject to substantial inter-individual variability, yet clinical practice relies on universal pressure targets derived from population-level guidelines. The Smart Inline Compliance Module (SICM) is a novel inline retrofit device that acquires intra-abdominal pressure and insufflation gas flow through physically separated sensing circuits, reconstructs insufflated volume by numerical integration of the flow signal, and derives the abdominal P–V curve and its biomechanical parameters in real time. This study reports the first two-arm pilot technical evaluation of the SICM system. Arm A comprised an exploratory biomechanical phantom with three defined stiffness levels (Soft, Medium, Rigid) tested under Continuous and Stepwise insufflation protocols (30 curves). Arm B comprised three female feline cadavers assessed under the same dual-protocol design (18 curves). This study should be interpreted as an early-stage technical evaluation rather than as a definitive validation benchmark. Signal quality was consistently high across both arms (Curve Quality Index: 1.0000 in the phantom arm; 0.9974 ± 0.0009 in the cadaveric arm). Volume integration accuracy was confirmed against an independent offline reference (mean absolute percentage difference: 0.07%). The system extracted reproducible biomechanical parameters under the Continuous protocol: in the cadaveric arm, maximum compliance (Cmax) ranged from 116.8 to 191.4 mL/mmHg across subjects, with intra-session coefficients of variation below 16%; Knee Pressure (Pknee), defined as a working operational index of the compliance transition, was 3.33–4.17 mmHg with CV below 8%. The Rigid phantom and cadaveric datasets showed partial numerical overlap in selected shape-derived parameters, which was interpreted only as an internal consistency check and not as evidence of biomechanical equivalence. The Stepwise protocol exposed the current methodological limits of the parameter-extraction workflow and identified specific targets for the next development iteration. These results are interpreted exclusively within the scope of technical feasibility and preliminary biomechanical characterisation; clinical applicability and optimal pressure guidance require adequately powered in vivo studies.

## Simple Summary

Laparoscopic surgery requires abdominal insufflation to a target pressure, but the mechanical response of the abdominal wall to gas delivery varies substantially between individuals. The Smart Inline Compliance Module (SICM) is an inline retrofit device designed to acquire the abdominal pressure–volume curve in real time during standard insufflation procedures, without modifying the insufflator or the surgical workflow. This pilot study evaluated the system in two preclinical experimental models of increasing biomechanical complexity—a biomechanical phantom and a feline cadaveric preparation—to assess its technical feasibility and its ability to extract reproducible biomechanical parameters. Results support the technical feasibility of the approach and provide a preliminary characterisation of the abdominal pressure–volume relationship across different mechanical conditions, laying the groundwork for further validation in biological models.

## 1. Introduction

Laparoscopy has become the standard of care for a broad range of surgical procedures in small animal veterinary medicine, with applications in dogs and cats expanding steadily over the past two decades [1,2]. Compared with open surgery, the laparoscopic approach offers well-documented advantages including reduced postoperative pain, shorter recovery time [3], and lower rates of surgical site infection [4]. However, the creation and maintenance of an adequate pneumoperitoneum requires CO_2_ insufflation to a target pressure, and this procedure is not without risk. The adverse haemodynamic and respiratory consequences of elevated intra-abdominal pressure (IAP) are well characterised across species [5,6,7], and are particularly relevant in small veterinary patients where the physiological reserve is limited. Beyond cardiorespiratory effects, splanchnic vascular consequences are also clinically significant: in a prospective clinical study in dogs, portal pressure increased by approximately 38% at 6 mmHg and by 175% at 14 mmHg compared to baseline, following an exponential model that supports the use of the minimum insufflation pressure necessary for adequate visualisation [8].

Current clinical guidelines for veterinary laparoscopy adopt pressure thresholds derived from human medicine, typically recommending IAP limits of 12–15 mmHg regardless of patient size or individual abdominal mechanics [9]. This approach is methodologically inconsistent with the known physiology of the abdominal pressure–volume (P–V) relationship. Abdominal compliance (Cab), defined as the change in intra-abdominal volume per unit change in IAP (mL/mmHg), is not a fixed parameter: it varies between individuals, changes dynamically during insufflation, and is determined by the combined elasticity of the abdominal wall, diaphragm, and visceral contents [10,11]. The P–V relationship is intrinsically curvilinear—initially near-linear at low pressures, then transitioning toward an exponential increase beyond a mechanical yield point above which each additional pressure increment generates diminishing workspace gain while imposing disproportionate mechanical stress on intra-abdominal tissues [12,13]. Applying a universal pressure target without knowledge of the individual P–V curve means that the same IAP may correspond to an efficient mechanical state in one patient and to post-yield overinsufflation in another. This variability is not negligible: Becker et al. (2017) demonstrated in human patients that abdominal wall compliance differs significantly between individuals and correlates exponentially with subcutaneous fat thickness, reinforcing the clinical rationale for patient-specific assessment over universal pressure targets [14].

The critical importance of individual P–V curve characterisation has been recognised in the literature, yet translating this concept into clinical practice has been constrained by the absence of validated real-time monitoring tools. Two foundational studies by Dorn and colleagues characterised the P–V curve in dogs and cats during capnoperitoneum using a stepwise syringe-based method, identifying a mean cutoff pressure (COP)—the yield point of the P–V curve—of 5.99 ± 0.81 mmHg in dogs [15] and 6.44 ± 1.7 mmHg in cats [16], with a wide inter-individual range (2.72–13.00 mmHg in cats) underscoring the need for patient-specific assessment. However, the syringe-based protocol is incompatible with routine clinical workflows: it requires dedicated equipment, offline data processing, and interruption of the standard insufflation procedure, precluding integration into daily practice. In the human literature, Sterke et al. (2022) proposed endoscopic oscillometry as a novel approach for monitoring Cab during laparoscopy, demonstrating feasibility in a porcine model (adjusted R^2^ = 97.1% vs CT-based compliance estimates), but the method requires a custom-built insufflator with dedicated oscillatory pressure generation circuitry and is not backward-compatible with standard equipment without hardware modification [17].

The technological gap—the absence of a validated, inline, real-time monitoring system compatible with standard insufflators—motivated the development of the Smart Inline Compliance Module (SICM), an external retrofit module that acquires intra-abdominal pressure and insufflation gas flow via physically separated dedicated sensing lines, reconstructs insufflated volume by numerical integration of the flow signal, and derives the abdominal P–V curve and its biomechanical parameters in real time, without modifying the insufflator or altering the surgical workflow. The long-term objective of the SICM platform is to support data-driven, patient-specific characterisation of abdominal biomechanics as a basis for individualised insufflation pressure guidance; the present study addresses the upstream question of whether the system can reliably acquire and characterise the abdominal P–V curve under controlled experimental conditions.

Before any clinical application can be considered, a technology of this nature must undergo rigorous methodological validation across progressively complex biomechanical models. The present study reports the first two-arm pilot technical evaluation of the SICM system, designed to assess signal acquisition, parameter extractability, and internal consistency across progressively more complex experimental substrates. Arm A is an exploratory phantom model comprising three nominal stiffness levels (Soft, Medium, Rigid) tested with two insufflation protocols (Continuous and Stepwise). Arm B is a feline cadaveric model comprising three subjects of varying body weight assessed under the same dual-protocol design. The primary endpoints are: (1) signal acquisition quality across all experimental conditions; (2) intra-session reproducibility of extracted biomechanical parameters; (3) preliminary discriminative sensitivity of the system across distinct mechanical conditions; and (4) system consistency between the phantom and cadaveric datasets as a prerequisite for transition to ex vivo and in vivo validation stages.

We hypothesised that the SICM system would be capable of acquiring high-quality pressure and flow signals across both experimental arms, that the Continuous insufflation protocol would yield reproducible biomechanical parameters with intra-session coefficients of variation consistent with an exploratory pilot design, and that the Rigid phantom and cadaveric datasets would show overlapping parameter ranges, supporting the internal consistency of the analytical workflow as a prerequisite for progression to subsequent validation stages.

## 2. Materials and Methods

### 2.1. Study Design

This study adopts a two-arm sequential technical-evaluation design. Arm A (Phantom Arm) used an exploratory biomechanical phantom as the first-level mechanical testbed, allowing characterisation of system performance under defined nominal stiffness conditions. Arm B (cadaveric model) used a feline cadaveric preparation as the second-level validation model, introducing biological complexity while maintaining an experimentally controlled environment. Both arms shared the same SICM hardware, acquisition protocols, and parameter-extraction workflow, supporting a harmonised parameter-based descriptive comparison across arms rather than a claim of biomechanical equivalence between the two substrates. The phantom arm was conducted first, prior to the cadaveric experiments, consistently with the progressive validation strategy adopted in this study.

### 2.2. SICM System—Hardware and Data Acquisition

The SICM (Smart Inline Compliance Module, Console v1.0) is based on an embedded microcontroller unit connected to a web browser-based graphical user interface (GUI v1.5.0). The system acquires intra-abdominal pressure via a high-resolution absolute pressure transducer (range 0–15 PSI) and insufflation gas flow via a calibrated mass flow sensor (range ±300 slm). A key architectural feature of the system is the physical separation of the pressure and flow sensing circuits: flow is measured inline between the insufflator and the access trocar, while pressure is measured via a dedicated secondary trocar connected to the pressure transducer by a sterile tube. This separation is designed to eliminate cross-interference between the dynamic flow signal and the quasi-static pressure signal. Both signals undergo onboard digital low-pass filtering prior to transmission to the graphical interface.

**Figure 1:**
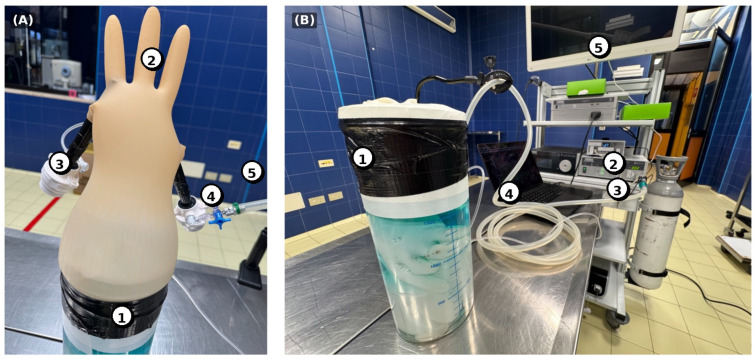
Biomechanical phantom benchmark setup. (**A**) Close-up view of the phantom model: (1) Hermetically sealed 3 L container body; (2) latex surgical glove membranes constituting the compliant outer wall (Rigid stiffness condition shown); (3) left trocar port connected to the SICM dedicated pressure-sensing line via sterile tube and stopcock; (4) right trocar port connected to the insufflation circuit incorporating the inline flow sensor; (5) stopcock assembly on the pressure line. (**B**) Overview of the complete experimental setup: (1) Phantom model on the surgical table; (2) SICM Console v1.0 connected via insufflation tubing to the phantom; (3) CO_2_ insufflator (Karl Storz Thermoflator); (4) laptop running the SICM graphical user interface (GUI v1.5.0); (5) laparoscopic tower in the background.

**Figure 2:**
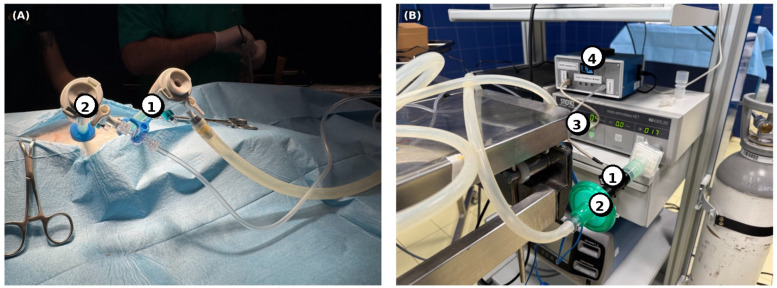
Cadaveric arm experimental setup. (**A**) Intraoperative view of trocar placement on a feline cadaver in dorsal recumbency: (1) Access trocar (5 mm) connected to the insufflation line incorporating the SICM inline flow sensor; (2) secondary trocar (5 mm) connected to the SICM dedicated pressure-sensing line via sterile tube and stopcock. (**B**) SICM hardware circuit on the instrument trolley: (1) Inline mass flow sensor (black housing) interposed between the insufflator outlet and the insufflation tubing; (2) hydrophobic bacterial filter on the pressure-sensing line inlet; (3) CO_2_ insufflator (Karl Storz endo-arthroflator-VET); (4) SICM Console v1.0 (blue unit) receiving pressure and flow signals and transmitting processed data to the GUI in real time.

Data were exported in CSV format at an effective sampling rate of approximately 15–16 Hz across all sessions, which was adequate for reconstruction of quasi-static P–V curves under both protocols tested (Figure 1 and Figure 2).

### 2.3. Insufflation Protocols

#### 2.3.1. Continuous Protocol

Continuous insufflation at constant flow rate (set flow: 1.5 L/min). Acquisition was terminated upon reaching the target pressure and plateau stabilisation (Q < 10 mL/s for ≥5 consecutive seconds in cadaveric trials; equivalent pressure plateau in phantom trials). Three consecutive sessions per subject or condition, with full system reset between trials.

#### 2.3.2. Stepwise Protocol

Insufflation in pressure steps of 2 mmHg, with a stabilisation plateau of at least 5 s between steps. Three consecutive sessions per subject or condition. Mean session duration was substantially longer than the Continuous protocol (cadaveric: 132 ± 22 s vs. 35 ± 7 s for Continuous).

### 2.4. Volume Integration—Cadaveric Arm

Cumulative insufflated volume V(t) was reconstructed by the SICM system from the continuous flow signal using numerical integration, with V initialised at zero at the start of each acquisition. The accuracy of the onboard volume reconstruction was validated by comparing system-derived V values against an independent reference integration computed offline across all 18 cadaveric curves. The mean absolute percentage difference between the two methods was 0.07% (maximum: 0.2%), confirming that the SICM volume integration algorithm operated with negligible computational error across all sessions. For phantom trials, volume was directly available in the exported data file; no integration step was required.

### 2.5. Biomechanical Parameter Extraction

The following analytical workflow was applied uniformly to all curves in both arms:

For conceptual reproducibility, the analytical sequence can be summarised as follows. After exclusion of non-active insufflation phases, pressure samples were grouped into narrow pressure bins along the ascending limb of the P–V curve. Within each bin, a representative volume value was computed to reduce point-to-point noise while preserving global curve morphology. Local compliance was then estimated as the discrete derivative of volume with respect to pressure, C(P) = ΔV/ΔP, along the binned curve. A smoothing step was applied to the resulting C(P) profile to attenuate high-frequency fluctuations before identifying PCmax and Cmax. Pknee was subsequently identified as the first pressure beyond PCmax at which the smoothed C(P) profile decreased below 75% of the session-specific Cmax. CQI was computed as a dimensionless pressure-signal regularity index based on the magnitude of local residual fluctuations relative to the global pressure trend, with values approaching 1.0 indicating a highly regular pressure trace. Exact implementation constants, including filter coefficients and embedded software thresholds other than the disclosed 75% Cmax criterion, are proprietary; however, the analytical logic, derived parameters, and interpretation rules are reported here to allow independent conceptual evaluation.

Identification of the active insufflation phase: samples corresponding to active gas delivery were selected based on pressure, volume, and—for cadaveric curves—flow rate thresholds. This step excludes the loss-compensation plateau phase, during which volume increases slowly at near-constant pressure without active delivery. For phantom curves, all ascending-phase samples were included.

Pressure-binned volume aggregation: The pressure axis was divided into discrete intervals, and the central tendency of volume within each interval was computed to reduce signal noise while preserving the overall P–V curve morphology.

Local compliance estimation: The discrete derivative of the aggregated volume with respect to pressure was computed to obtain a local compliance function C(P) along the curve.

Smoothing of C(P) to attenuate residual high-frequency noise while preserving biomechanically relevant transitions.

Identification of Cmax as the maximum value of C(P) within the active pressure range, and of PCmax as the corresponding pressure.

Identification of Knee Pressure (Pknee) as the pressure beyond PCmax at which C(P) falls below a defined fractional threshold relative to Cmax, indicating the onset of the compliance reduction phase.

### 2.6. Derived Parameters

The following biomechanical parameters were derived for each curve:

**PCmax (mmHg):** Pressure at maximum compliance—the insufflation pressure at which the P–V curve exhibits maximum system expansibility.

**Cmax (mL/mmHg)**: Maximum compliance observed along the P–V curve during the active insufflation phase.

**Pknee (mmHg):** Knee Pressure—a working operational index of the biomechanical compliance transition, defined as the first pressure beyond PCmax at which C(P) falls below 75% of the individual session Cmax (i.e., a 25% fractional drop threshold). This threshold was selected on the basis of two operational criteria: (i) robustness to intra-session noise in the compliance profile, which requires a threshold large enough to avoid spurious detection from minor C(P) fluctuations inherent in biological signals; and (ii) suitability for real-time use, which requires a threshold that can be evaluated incrementally as the curve is acquired without requiring post hoc fitting or global curve knowledge. A 25% fractional threshold represents a pragmatic balance between these two requirements. No formal threshold optimisation was performed in this pilot study; sensitivity analysis across alternative thresholds (e.g., 20%, 30%) is planned for future validation work with larger datasets, where the impact on Pknee estimates and their clinical relevance can be formally assessed. Pknee is a device-specific operational metric. Importantly, Pknee should not be interpreted as a physiologically validated transition pressure or as a patient-specific safety threshold. In the present implementation, it is an algorithm-derived descriptor of the post-PCmax compliance decline according to the SICM analytical framework. Its biological meaning, threshold sensitivity, and relationship with independently derived P–V transition points require dedicated validation in larger ex vivo and in vivo datasets.

**Work Zone (mmHg)**: In this first analytical iteration, the Work Zone was operationally defined as the interval between PCmax and Pknee, representing the pressure range immediately following the point of maximum local compliance and preceding the predefined compliance decline threshold. This construct is intended as a preliminary, device-specific descriptor of the mechanically efficient portion of the P–V curve. It should not be interpreted as a validated clinical target or safety boundary, and its definition may require refinement in future datasets as more complex compliance-curve morphologies are characterised.

**MaxP (mmHg):** Maximum pressure reached during the session.

**MaxV (mL):** Maximum insufflated volume during the session.

**Slope (mmHg/mL):** Mean slope of the P–V curve computed by linear regression over the active insufflation phase (cadaveric arm only).

**CQI (dimensionless):** Curve Quality Index—a signal smoothness metric derived from the statistical properties of local residuals in the acquired pressure trace. Values approaching 1.0 indicate high signal regularity; lower values reflect greater high-frequency noise. This index is more informative in the cadaveric arm, where dynamic flow introduces real signal variation, than in the passive phantom setting where it approaches its theoretical maximum by design.

Specific implementation details of the parameter-extraction analytical workflow—including signal conditioning parameters, pressure-binning intervals, smoothing algorithms, and the Curve Quality Index computation—are not fully disclosed in this manuscript, as they constitute proprietary technical elements covered by European Patent Application No. EP26164347.2. The published results are fully reproducible at the level of the described experimental protocol and outcome parameters; the proprietary elements concern the embedded computational implementation and are not required for independent validation of the reported biomechanical findings.

A high-level schematic overview of the analytical workflow, illustrating the sequential processing steps and their logical dependencies, is provided in Supplementary Figure S1. This flowchart describes the structural logic of the analytical workflow without disclosing the specific implementation parameters and algorithms covered by European Patent Application No. EP26164347.2.

### 2.7. Statistical Analysis

This was an exploratory, descriptive, two-arm preclinical validation study. Arm A comprised a biomechanical phantom tested under three stiffness conditions and two insufflation protocols; Arm B comprised three feline cadaveric subjects assessed under the same dual-protocol design. Each condition was tested with three consecutive acquisitions per subject or group. No randomisation, blinding, or concurrent control group was included. The sample size was not based on a formal power calculation but is consistent with the scope of a first-level exploratory pilot validation. All statistical procedures described below are descriptive in nature; no inferential hypothesis testing was performed.

Intra-subject variability (cadaveric arm) and intra-group variability (phantom arm) were quantified using standard deviation (SD) and percentage coefficient of variation (CV%) computed across the three repeated measures per subject or condition per protocol. CV% values were reported descriptively as indicators of intra-session variability. Given the exploratory design and the limited number of repeated acquisitions, no qualitative robustness categories were used to infer statistical strength.

A sample of *n* = 3 per subject (cadaveric) or *n* = 5–6 per stiffness–protocol combination (phantom) is consistent with an exploratory pilot study design and does not support formal inferential statistical testing with adequate power. All comparisons reported in this study are therefore descriptive in nature. Results are expressed as mean ± standard deviation.

In the cadaveric arm, the three repeated curves per subject per protocol are more precisely characterised as within-subject technical replicates—repeated measures on the same biological individual—rather than biologically independent observations. This distinction is methodologically relevant: the curves document intra-session technical reproducibility of the system within a given subject, but do not support inference about inter-subject or population-level variability. Any between-subject comparison in this study is purely descriptive and exploratory.

The experimental unit for all analyses is the individual curve (cadaveric arm: *n* = 3 technical replicates per subject per protocol; phantom arm: *n* = 5–6 per stiffness–protocol cell). Samples acquired at 15–16 Hz do not constitute independent statistical replicates: the high temporal resolution improves curve reconstruction quality but does not increase the number of independent observations. Any analysis treating individual time-series samples as replicates would constitute pseudoreplication.

All descriptive statistics (mean, standard deviation, CV%) were computed using custom Python scripts (Python 3.11.9; NumPy 2.4.4; pandas 3.0.2).

### 2.8. Phantom Model—Arm A

The phantom benchmark was conducted using a hermetically sealed 3 L container configured to mechanically simulate the abdominal cavity of a small animal (≤10 kg). The phantom is not intended as an anatomical replica of the abdomen, but as a controlled mechanical system with defined and reproducible deformability characteristics and pressure response. The interior was filled with differentiated materials to reproduce the mechanical heterogeneity of abdominal compartments: ultrasound gel for parenchymal compartments, water-filled bags for fluid structures, and an inflated latex surgical glove for the gaseous compartment. The outer wall was constructed using surgical gloves as membranes of three defined stiffness levels: Soft, Medium, and Rigid. All trials were conducted in a closed system starting from atmospheric pressure. A total of 30 unique curves were acquired, curves were considered valid for statistical analysis if all of the following criteria were met: (i) CQI ≥ 0.990; (ii) MaxV within 2.5 SD of the respective stiffness–protocol group mean; and (iii) absence of documented hardware events (sensor disconnection, tubing displacement, or seal failure) during acquisition. Curves failing any criterion were excluded from statistical analysis and reported separately. Applying these criteria, two curves were excluded, S11 (Medium, Stepwise) and M11 (Soft, Continuous), both presenting MaxV values approximately 3–6× the respective group mean in the absence of concurrent CQI or MaxP anomalies, consistent with a non-standard phantom configuration during those specific sessions (see Section 3.1.5). The 28 remaining curves constituted the valid dataset for all statistical analyses. The phantom should therefore be interpreted as an exploratory mechanical testbed rather than as a fully standardised reference phantom. Although nominal stiffness conditions were predefined, small variations in membrane positioning, filling distribution, wall constraint, and trocar/tubing configuration could influence volume accommodation. This limitation is directly reflected by the occurrence of two volumetric outliers despite preserved pressure-signal regularity. The Rigid phantom was acquired exclusively with the Continuous protocol (*n* = 6). Both protocols are available for Soft (Continuous *n* = 5, Stepwise *n* = 6) and Medium (Continuous *n* = 6, Stepwise *n* = 5). The Rigid group was primarily intended to provide a phantom condition with a detectable compliance transition and a P–V morphology closer to that observed in the cadaveric arm, thereby serving as a reference condition for assessing the internal consistency of the analytical workflow across experimental substrates. For this purpose, the Continuous protocol was selected because it provides a single quasi-monotonic ascending curve and therefore allows the most direct comparison with the cadaveric Continuous acquisitions. Stepwise characterisation of the Rigid condition was considered outside the scope of this first validation stage and is deferred to subsequent validation iterations.

### 2.9. Cadaveric Model—Arm B

The cadaveric arm was conducted on three female feline cadavers (G1: 3.0 kg; G2: 4.0 kg; G3: 3.2 kg) that had undergone euthanasia for clinical conditions unrelated to the study protocol. All cadavers were used within 60 min of euthanasia, and stored at 18–20 °C. None of the subjects had a history or macroscopic evidence of abdominal trauma, thoracic trauma, or abdominal conditions expected to substantially alter abdominal wall mechanics. Cadavers were positioned in dorsal recumbency. After routine clipping and antiseptic preparation of the ventral abdomen, two 5 mm trocars were placed on the linea alba using a standard Hasson open technique. The first trocar was positioned in the subumbilical region and connected to the insufflation line through the SICM inline flow-sensing circuit. The second trocar was positioned cranially, approximately midway between the umbilicus and the xiphoid process, and connected to the dedicated SICM pressure-sensing line through a sterile tube and stopcock. This trocar configuration was selected to reproduce a simplified access geometry comparable to that used during feline laparoscopic ovariectomy. Before each acquisition, pneumoperitoneum integrity was checked by confirming stable pressure maintenance during low-flow insufflation. Sessions with suspected loss of pneumoperitoneum integrity were flagged and excluded from analysis when appropriate, as described for the volumetric outlier in Section 3.2.6. Three consecutive acquisition sessions per subject per protocol were performed, with full system reset between trials, for a total of 18 curves across both protocols.

## 3. Results

### 3.1. Arm A—Phantom Benchmark

#### 3.1.1. Signal Quality

The CQI reached 1.0000 in all 30 phantom curves across all stiffness levels and protocols (CV = 0.0%). As detailed in Section 2.6, the CQI is constructed as a smoothness metric of the pressure signal; in a closed passive mechanical system without dynamic flow variation, the pressure signal is intrinsically smooth and the index approaches its theoretical maximum by design. The CQI is therefore reported here primarily for cross-arm comparability and should not be interpreted as evidence of discriminative quality in the phantom context.

#### 3.1.2. Biomechanical Parameters by Stiffness and Protocol

Biomechanical parameters are reported in Table 1. For the Rigid group (Continuous protocol, *n* = 6): PCmax = 2.75 ± 0.63 mmHg; Cmax = 139 ± 18 mL/mmHg; Pknee = 4.08 ± 0.73 mmHg (Work Zone: 1.33 ± 0.37 mmHg); MaxV = 1357 ± 63 mL; MaxP = 15.10 ± 0.22 mmHg. For Soft and Medium groups, PCmax was consistently located around 10.2–10.5 mmHg in both protocols, and Pknee was not detectable within the acquired pressure range (~11–12 mmHg).

#### 3.1.3. Intra-Group Variability

Table 2 reports CV% values for each stiffness × protocol combination. For the Rigid group: Cmax CV = 13.3%; Pknee CV = 17.9%; MaxV CV = 4.7%. For Soft, the Stepwise protocol produced Cmax CV = 22.6% versus 50.9% for Continuous. For Medium, the Continuous protocol produced Cmax CV = 18.1% versus 54.4% for Stepwise. MaxP and PCmax showed CV < 5% across all conditions.

#### 3.1.4. Protocol Comparison—Phantom

For the Soft group, Cmax was nearly identical between protocols (714 ± 363 vs. 702 ± 158 mL/mmHg; Δ = −1.6%), with a marked difference in variability (CV 50.9% vs. 22.6%). MaxV was 9.7% higher in the Stepwise protocol (4471 ± 260 vs. 4077 ± 315 mL). For Medium, Cmax differed substantially between protocols (366 ± 66 Continuous vs. 730 ± 397 mL/mmHg Stepwise; Δ = +99.6%), with the Continuous protocol being more stable (CV 18.1% vs. 54.4%). MaxV was 42.2% higher in Stepwise (4993 vs. 3512 mL). MaxP was invariant between protocols across all conditions (Δ < 2%).

#### 3.1.5. Outliers—S11 and M11

Two phantom curves were excluded from statistical analysis. S11 (Medium, Stepwise): Cmax = 2244 mL/mmHg (~6× the group mean), MaxV = 6057 mL. M11 (Soft, Continuous): Cmax = 2109 mL/mmHg (~3× the group mean), MaxV = 5253 mL. In both cases CQI remained 1.0000 and MaxP was within the normal range, indicating that the pressure signal was correct. The anomaly is attributed to a non-standard phantom configuration during those specific sessions. The occurrence of these outliers reflects an inherent limitation of the current phantom benchmark design: the phantom configuration was not rigidly standardised across all sessions, and this represents a source of variability that limits its claim as a perfectly controlled reference model.

### 3.2. Arm B—Cadaveric Model

#### 3.2.1. Signal Quality and Volume Validation

The global mean CQI across all 18 cadaveric curves was 0.9974 ± 0.0009 (range: 0.9955–0.9984). The Stepwise protocol yielded a marginally higher CQI than the Continuous protocol (0.9981 ± 0.0003 vs. 0.9968 ± 0.0009), attributable to the smoother pressure profile during equilibration plateaux. Intra-subject CQI variability was below 0.1% for all subjects and protocols. In contrast to the phantom arm, the cadaveric CQI reflects genuine signal variation introduced by dynamic insufflation against a viscoelastic wall, and is therefore a meaningful quality metric in this context. Volume validation confirmed a very small difference between firmware-integrated and reference offline integration values: mean absolute percentage difference 0.07%, maximum 0.2% across 18 curves.

#### 3.2.2. Biomechanical Parameters—Continuous Protocol

**Figure 3:**
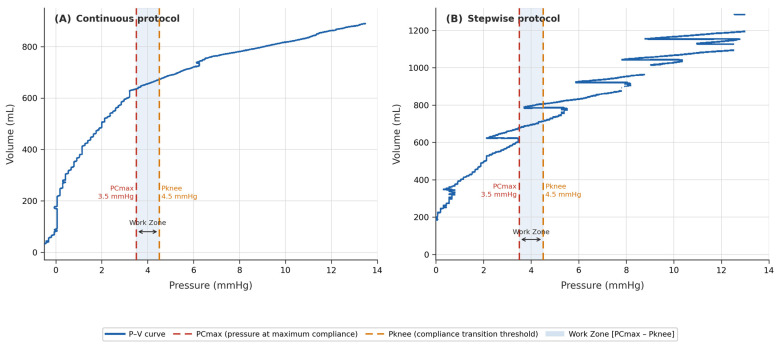
Representative abdominal pressure–volume (P–V) curves acquired in the same feline cadaveric subject under the two insufflation protocols. (**A**) Continuous protocol (constant flow: 1.5 L/min): The curve displays a smooth, quasi-monotonic ascending profile, enabling reliable extraction of all biomechanical parameters. PCmax = 3.5 mmHg; Pknee = 4.5 mmHg; Work Zone = 1.0 mmHg. (**B**) Stepwise protocol (2 mmHg steps with ≥5 s equilibration plateaux): The characteristic staircase morphology reflects successive delivery-to-plateau transitions; viscoelastic tissue relaxation at each step produces a systematically higher final volume compared to the Continuous protocol (+27.9% MaxV). PCmax = 3.5 mmHg; Pknee = 4.5 mmHg. The shaded area indicates the operational Work Zone descriptor [PCmax–Pknee], operationally defined in this first analytical iteration as a preliminary candidate pressure-efficiency descriptor.

Biomechanical parameters for the Continuous protocol are summarised in Table 3. PCmax was 2.0 mmHg in both G1 and G3 (CV < 1%), and 3.0 ± 0.4 mmHg in G2 (CV = 13.6%). Cmax showed low intra-subject variability in G1 and G3, and higher variability in G2: 157.9 ± 5.8 mL/mmHg in G1 (CV = 3.7%), 191.4 ± 13.9 mL/mmHg in G3 (CV = 7.3%), and 116.8 ± 17.7 mL/mmHg in G2 (CV = 15.2%). Pknee showed the lowest intra-subject CV: CV < 1% for G1 (3.50 ± 0.00 mmHg) and <8% for G2 and G3 (4.17 ± 0.24 and 3.33 ± 0.24 mmHg, respectively). Weight normalisation revealed a notably distinct pattern in G2: Cmax/BW was 29.2 ± 4.4 mL/mmHg/kg, approximately half that of G1 (52.6 ± 1.9 mL/mmHg/kg) and G3 (59.8 ± 4.3 mL/mmHg/kg) (Figure 3).

#### 3.2.3. Intra-Subject Variability—Protocol Comparison

Table 4 summarises CV% values for key parameters across both protocols. G2 Stepwise is excluded for parameter unreliability (see Section 3.2.5).

**Table 4:** Intra-subject variability (CV%)—cadaveric arm.

Parameter	G1-Cont	G2-Cont	G3-Cont	G1-Step	G3-Step
PCmax (CV%)	<1%	13.6%	<1%	16.3%	33.1%
Cmax (CV%)	3.7%	15.2%	7.3%	9.6%	24.4%
Pknee (CV%)	<1%	5.7%	7.1%	<1%	32.7%
MaxV (CV%)	14.1%	12.5%	5.7%	5.9%	1.0%
Slope (CV%)	10.4%	20.9%	11.2%	7.6%	0.8%
CQI (CV%)	0.1%	0.0%	0.1%	0.0%	0.0%

G2 Stepwise: Cmax and Pknee unreliable—excluded. MaxV reported separately in Table 5.

**Table 5:** Biomechanical parameters—cadaveric arm, Stepwise protocol (mean ± SD). * G2 Stepwise: Parameters not reliably extractable with the current analytical workflow. † Excludes S_G2_T3 (volumetric outlier, MaxV = 3574 mL; probable trocar seal failure). — = parameter not computable.

Parameter	G1 (3.0 kg)	G2 (4.0 kg) *	G3 (3.2 kg)
PCmax (mmHg)	2.50 ± 0.41	—	6.33 ± 2.09
Cmax (mL/mmHg)	169.4 ± 16.2	—	239.3 ± 58.3
Pknee (mmHg)	3.50 ± 0.00	—	7.50 ± 2.45
MaxV (mL)	1134 ± 67	1502 ± 230 †	1527 ± 15
MaxP (mmHg)	15.93 ± 0.45	14.75 ± 0.05	17.23 ± 1.17
+MaxV vs. cont. (%)	+27.9%	+40.4% †	+34.4%

#### 3.2.4. Inter-Subject Comparison—Continuous Protocol

G1 and G3 exhibited similar biomechanical profiles: identical PCmax (2.0 mmHg), closely matched Pknee (3.50 vs. 3.33 mmHg), and comparable proposed work zones (1.50 vs. 1.33 mmHg). The primary difference was absolute Cmax (157.9 vs. 191.4 mL/mmHg), potentially reflecting anatomical differences in abdominal cavity volume or visceral filling state. G2 exhibited a consistently lower Cmax/BW and marginally higher Pknee. MaxV/BW differed across subjects (267–355 mL/kg) without a simple correlation with body weight. These inter-subject differences are descriptive observations on a three-subject pilot dataset and should not be extrapolated to population-level conclusions.

#### 3.2.5. Stepwise Protocol—Results

Stepwise protocol results are summarised in Table 5.

For G1, the Stepwise protocol yielded technically reproducible parameters consistent with the Continuous protocol: Cmax = 169.4 ± 16.2 mL/mmHg (CV = 9.6%), Pknee = 3.50 ± 0.00 mmHg (CV < 1%), indicating that the analytical workflow is functional under stepwise loading in subjects with regular P–V profiles. For G3, Stepwise Pknee was higher than Continuous (7.50 ± 2.45 vs. 3.33 ± 0.24 mmHg; CV = 32.7%), a discrepancy that serves as a methodologically useful signal rather than an anomaly: it indicates that under slow step-equilibrated insufflation, viscoelastic tissue relaxation shifts the compliance transition point in a manner that the current analytical workflow may not fully resolve. For G2, Cmax and Pknee could not be reliably extracted, with computed Cmax values of 580 and 219 mL/mmHg and PCmax at 11–12 mmHg—biomechanically implausible results indicating that the Stepwise P–V profile of this subject, characterised by large flow oscillations and rapid delivery-to-plateau transitions, exceeded the tested pressure range of the current parameter-extraction pipeline. MaxV remains the most robust parameter under Stepwise conditions. Taken together, the Stepwise results are informative precisely because they identify the methodological limits of the current analytical workflow, providing a structured basis for targeted methodological improvements in subsequent iterations.

#### 3.2.6. Volumetric Outlier—S_G2_T3

The third Stepwise trial of G2 produced MaxV = 3574 mL (~2.8 SD above the valid curve mean). Firmware-integrated and reference-integrated volumes agreed within 0.04%, excluding a computational artefact. The probable cause is loss of pneumoperitoneum integrity (trocar displacement or seal failure). This trial was excluded from all analyses.

#### 3.2.7. Protocol Comparison—Volume and Duration

The Stepwise protocol produced systematically higher MaxV than Continuous in all three subjects: G1 +27.9% (1134 ± 67 vs. 886 ± 125 mL); G2 +40.4% (1502 ± 230 vs. 1070 ± 134 mL, excluding outlier); G3 +34.4% (1527 ± 15 vs. 1137 ± 64 mL). The two protocols are not interchangeable for volumetric comparisons. Mean session duration was substantially longer for Stepwise (132 ± 22 s vs. 35 ± 7 s for Continuous).

### 3.3. Cross-Arm Parameter Consistency: Rigid Phantom vs. Cadaveric Model

To evaluate the internal coherence of the SICM analytical workflow across physically distinct substrates, key biomechanical parameters from the Rigid phantom group (Continuous protocol, *n* = 6) were compared with those from the feline cadaveric arm (Continuous protocol). This comparison is strictly an internal consistency check—it addresses whether the same pipeline produces structurally comparable outputs when applied to substrates with similar P–V profile morphology—and does not imply biological equivalence between a sealed mechanical container and a cadaveric abdominal cavity. Parameters are summarised in Table 6.

PCmax, Pknee, Work Zone width, and MaxP showed numerical overlap between the Rigid phantom and cadaveric subjects across both G1 + G3 and G2 subgroups. The systematic excess in phantom MaxV (+30–34%) relative to the cadaveric arm is mechanically expected, given the absence of gas leakage and peritoneal CO_2_ absorption in the sealed system, and does not affect the comparability of shape-derived parameters. Taken together, these data indicate that the analytical consistency produces consistent parameter estimates across substrates with qualitatively similar P–V profiles, which is a necessary prerequisite for progression to ex vivo and in vivo validation stages.

## 4. Discussion

The results reported in this study are interpreted exclusively within the scope of technical feasibility and preliminary biomechanical characterisation. No inference is drawn regarding clinical applicability, optimal pressure guidance, or superiority to existing methods. These questions require adequately powered in vivo studies with live subjects and constitute later stages of this validation analytical consistency, not outcomes of the present work.

The CQI data across both arms merit a careful, differentiated interpretation. In the cadaveric arm, the mean CQI of 0.9974 ± 0.0009 reflects genuine signal quality under dynamic insufflation conditions: variable flow rates against a viscoelastic biological wall introduce real pressure fluctuations that the CQI detects, and the consistently high values confirm that the SICM signal conditioning analytical consistency attenuates this noise while preserving the quasi-static biomechanical information of the P–V curve. In the phantom arm, the CQI of 1.0000 reflects the intrinsic smoothness of pressure acquisition in a closed passive mechanical system, where no dynamic flow variation is present. As noted in the Methods, the CQI approaches its theoretical maximum in this setting by design, and its phantom value should be read as a boundary condition rather than as a discriminative quality signal. The meaningful CQI comparison is therefore within the cadaveric arm—between subjects and protocols—where it documents robust and reproducible acquisition independent of inter-individual or inter-session variation.

The stability of MaxP (CV < 2% across all conditions) and PCmax (CV < 3%) provides more robust evidence of system consistency: these parameters depend on the actual P–V curve structure and are not susceptible to the ceiling effect that limits CQI interpretation in the phantom context.

The results of this pilot study provide preliminary evidence that the SICM system is capable of distinguishing different biomechanical conditions at two complementary levels. In the phantom arm, the three stiffness conditions produced structurally distinct P–V profiles consistently separated across measured parameters: MaxP (~11.5 mmHg for Soft and Medium vs. ~15.1 mmHg for Rigid; Δ = +31%), MaxV (~4100–4400 mL vs. ~1357 mL; ratio approximately 3:1), and the presence or absence of a detectable Pknee. The absence of Pknee in Soft and Medium curves is a mechanically faithful result: these substrates maintain high compliance throughout the acquisible pressure range without a sharp transition, which the system correctly identifies as absence rather than a spurious detection.

The non-detectability of Pknee in the Soft and Medium phantom conditions is interpreted as a mechanically meaningful finding rather than as a failure of the extraction analytical consistency. In these highly compliant configurations, the C(P) profile remained elevated and did not show a sufficiently marked post-peak decline within the acquired pressure range (~11–12 mmHg). Consequently, the predefined 25% reduction from Cmax required for Pknee detection was not reached. This may reflect both the gradual compliance decay of the Soft and Medium membranes and the pressure range explored in the present protocol, which may have ended before a clear post-yield phase developed. In addition, the phantom is a simplified mechanical model that lacks several anatomical constraints present in biological abdomens, including heterogeneous visceral packing, diaphragmatic coupling, and thoracoabdominal boundary effects. Therefore, the absence of a detectable Pknee in these phantom conditions should be interpreted as indicating that no distinct compliance transition occurred within the tested range, rather than as evidence that such a transition cannot occur at higher pressures or in biological subjects.

It should be acknowledged that the phantom model was designed to provide a mechanically defined and reproducible compliance substrate for first-level analytical consistency validation, not to reproduce the anatomical topography of the abdominal cavity. In an intact abdomen, the spatial arrangement of the viscera, organ-to-organ contact, visceral filling state, diaphragmatic coupling, and abdominal wall geometry all contribute to the morphology of the P–V curve. These topographical and anatomical determinants cannot be fully reproduced by a simplified non-anatomical phantom. This distinction is important when interpreting the cross-arm comparison: the overlap observed between the Rigid phantom and cadaveric P–V-derived parameters indicates convergence of global mechanical behaviour under the tested conditions, but does not imply anatomical or biological equivalence. The progressive inclusion of ex vivo and in vivo biological substrates in subsequent validation stages will therefore be necessary to characterise the contribution of visceral topography to the abdominal P–V relationship and to assess how SICM performance translates to anatomically realistic conditions.

The phantom findings should not be interpreted as evidence that the device has been validated against a mechanically invariant reference standard. Rather, they demonstrate that the system can acquire and process P–V data across deliberately different mechanical configurations, while also revealing the sensitivity of the experimental setup to configuration-dependent variability.

In the cadaveric arm, the three subjects showed descriptively distinct biomechanical profiles under the Continuous protocol. Specifically, Cmax/BW ranged from 29.2 ± 4.4 mL/mmHg/kg in subject G2 to 59.8 ± 4.3 mL/mmHg/kg in subject G3, while Pknee showed good intra-subject technical reproducibility (CV < 8% in all three subjects) but differed between subjects (3.33–4.17 mmHg). These findings suggest that the SICM system can capture subject-specific differences in abdominal mechanical behaviour, although the present pilot dataset does not allow quantification of discriminative accuracy or population-level variability.

The PCmax values observed in the cadaveric Continuous protocol (2.0–3.0 mmHg) correspond to the insufflation pressure at which the abdominal cavity exhibits its maximum local compliance. These values are consistent with the expected behaviour of the feline abdominal wall in the early reshaping phase of insufflation, as conceptually described by Malbrain et al. (2014), in which volume gain per unit pressure increment is largest before elastic wall resistance begins to dominate [11]. From an analytical perspective, PCmax may represent a particularly informative descriptor because it is derived directly from the local maximum of the compliance function C(P), rather than from a predefined fractional decline threshold. In this sense, PCmax identifies the pressure at which the abdominal compartment expresses its greatest volume gain per unit pressure increase under the tested loading condition. In the present pilot study, PCmax is interpreted only as a candidate biomechanical descriptor and not as a clinical target. However, its stability under the Continuous protocol and its direct relationship with the local morphology of the P–V curve support its inclusion as a primary parameter for future validation studies aimed at refining pressure-efficiency descriptors of abdominal insufflation.

Pknee (3.33–4.17 mmHg in the cadaveric Continuous protocol) is defined operationally as the pressure at which compliance drops by 25% relative to the individual session Cmax. It is important to be explicit about the nature of this definition: Pknee is a working operational threshold, chosen for robustness and suitability for real-time use rather than derived from a biomechanically validated criterion. Its conceptual analogy with the cutoff pressure (COP) of Dorn et al. [15,16] is suggestive—both parameters aim to identify the upper boundary of the efficient insufflation zone—but the two indices are algorithmically distinct and quantitatively non-equivalent. The Dorn COP is derived geometrically from the exponential portion of the P–V curve and requires insufflation well beyond the transition point. SICM Pknee is derived from the ascending compliance profile and is available earlier in the insufflation sequence. Whether the two indices converge on the same physiological threshold in the same subjects under the same conditions is a question that remains open and constitutes a primary objective of future validation work. Sensitivity analysis of Pknee to threshold variation (e.g., 20% vs. 25% vs. 30% fractional drop) was not performed in this pilot study, as the three-subject cadaveric dataset does not provide sufficient statistical basis for a robust threshold optimisation. This analysis is identified as a methodological priority for the next validation stage, where a larger and biologically more representative dataset will allow formal characterisation of threshold-dependent Pknee variability and its relationship to clinically relevant compliance transition points.

The proposed SICM-defined Work Zone—operationally defined in this first analytical iteration as the interval [PCmax, Pknee]—ranged from 1.17 to 1.50 mmHg across cadaveric subjects and was 1.33 ± 0.37 mmHg in the Rigid phantom. In the present dataset, pressures within this interval corresponded to the early post-PCmax portion of the curve, where compliance remained close to its maximum before reaching the predefined decline threshold. This interval should therefore be interpreted as a preliminary pressure-efficiency descriptor rather than as a validated clinical target or safety boundary. Importantly, the Work Zone definition used here is intentionally conservative and may require refinement as larger datasets allow more detailed characterisation of compliance-curve morphology. Whether this or other PCmax-centred descriptors translate into clinically meaningful workspace optimisation requires prospective testing in adequately powered in vivo studies and cannot be inferred from the present data.

The distinct pattern of G2—Cmax/BW approximately half that of G1 and G3, and consistently reproduced across all three Continuous sessions—is compatible with greater relative abdominal wall stiffness in this subject. This descriptive observation illustrates the type of inter-individual difference that a patient-specific compliance monitoring system would need to capture, and motivates the expansion of the cadaveric cohort in subsequent studies.

The Stepwise protocol produced systematically higher MaxV than Continuous across all subjects and stiffness conditions, consistent with the viscoelastic behaviour of biological and biomechanical tissues under slow step-equilibrated loading [5,18]. This volumetric difference is a real mechanical effect, not an analytical artefact, and it means the two protocols are not interchangeable for volumetric comparisons.

The substantially longer session duration of the Stepwise protocol in the cadaveric arm (132 ± 22 s vs. 35 ± 7 s for Continuous) deserves specific consideration. Although Stepwise acquisitions produced higher final insufflated volumes, this did not translate into superior stability or broader extractability of derived biomechanical parameters. The longer duration mainly reflects the mandatory equilibration plateaux at each pressure step, which allow time-dependent viscoelastic relaxation and may explain the systematically higher MaxV values observed with this protocol. However, these same delivery-to-plateau transitions increase the complexity of the P–V profile and of the derived C(P) curve, limiting the robustness of Cmax and Pknee extraction with the current analytical workflow. From a practical perspective, the Continuous protocol provided more consistent parameter extraction in a substantially shorter acquisition time, which is directly relevant for future clinical workflow integration, where minimising unnecessary insufflation time is desirable. In the current system iteration, the Stepwise protocol should therefore be interpreted primarily as a research and benchmark modality rather than as the preferred acquisition protocol for real-time parameter extraction.

From a parametric stability perspective, the Continuous protocol yielded more consistent Pknee and Cmax values in the cadaveric arm. The Stepwise protocol was informative in a different sense: it functioned well in G1, where the P–V profile was sufficiently regular, but revealed the operating limits of the current parameter-extraction workflow in G2 (implausible Cmax values) and highlighted a protocol-dependent shift in Pknee in G3 (3.33 vs. 7.50 mmHg). Rather than treating these as analytical failures, the Stepwise results should be interpreted as structured boundary-condition data that helped identify the operating limits of the current parameter-extraction workflow. Two specific limitations were recognised. First, under complex multi-phase flow dynamics, as observed in G2 Stepwise, the rapid alternation between active delivery and equilibration plateaux generated a P–V profile with repeated local compliance fluctuations at successive pressure steps. The current analytical workflow was primarily designed for quasi-monotonic ascending curves under continuous loading and does not yet include a dedicated segmentation of active-delivery and plateau phases. Consequently, the implausible parameter estimates obtained in G2 Stepwise are consistent with inappropriate attribution of step-related local fluctuations to global compliance maxima. Second, even in subjects with more regular Stepwise profiles, as observed in G3, slow step-equilibrated loading allows progressive viscoelastic relaxation during each plateau, which may shift the apparent compliance transition toward higher pressures relative to continuous loading. This explains why Pknee values derived from Stepwise and Continuous protocols should not be interpreted as directly interchangeable.

These findings define specific targets for the next analytical workflow iteration. Planned modifications include (i) a dedicated step-segmentation module to distinguish active-delivery and plateau phases during Stepwise acquisitions; (ii) protocol-aware parameter extraction, with separate detection logic for continuous and step-equilibrated loading; and (iii) plausibility checks to flag parameter estimates that are inconsistent with the observed P–V morphology or with expected mechanical ranges for the tested model. On this basis, the Continuous protocol is retained as the reference modality for biomechanical parameter extraction in the current system iteration, whereas the Stepwise protocol is considered primarily a benchmark and stress-test modality until protocol-specific extraction tools are implemented.

The cross-arm consistency analysis (Section 3.3, Table 6) demonstrated numerical overlap in shape-derived parameters—PCmax, Pknee, Work Zone, and MaxP—between the Rigid phantom and cadaveric datasets, while the volumetric excess of the phantom (+30–34% MaxV) is mechanically expected and does not compromise parameter comparability. This observation should be interpreted conservatively as an internal consistency check showing that the same analytical workflow can return comparable shape-derived descriptors in two mechanically distinct substrates. It does not establish biological equivalence, model validity, or clinical transferability.

Feline values report G1 + G3 (*n* = 6) and G2 (*n* = 3) separately to document descriptive inter-subject differences. Phantom values are group means. This comparison is a consistency check, not a validation of biological equivalence.

The scope of this pilot study is explicitly limited to technical feasibility: demonstrating that the SICM system can acquire, process, and reproduce biomechanical P–V parameters under controlled conditions. The results are consistent with this scope. The system shows stable intra-session technical reproducibility for key parameters under the Continuous protocol, preliminary evidence of sensitivity to different mechanical conditions across both arms, and coherent behaviour across physically distinct substrates. What the results do not establish is clinical utility at this stage, optimal pressure guidance, or superiority to existing methods. These questions require in vivo validation with adequate sample sizes, and they represent the next steps of the validation analytical consistency rather than outcomes of the present work.

Several limitations must be explicitly acknowledged. First, the cadaveric sample (N = 3 subjects) is a minimal pilot dataset. All between-subject comparisons are descriptive, and the three-subject cohort is not representative of the biological variability of the feline population. Second, the three curves per subject per protocol are within-subject technical replicates, not biologically independent observations; the CV% values reported therefore quantify intra-session measurement reproducibility, not biological variability. Third, the cadaveric model does not replicate in vivo physiological conditions: the absence of muscle tone, respiratory movement, and anaesthesia-related pharmacological effects on abdominal wall mechanics may substantially modify both the P–V curve shape and the position of compliance transition points relative to live subjects [1,5]. Fourth, the phantom benchmark, while providing a useful first-level mechanical testbed, is not a perfectly standardised reference system: the occurrence of configuration-dependent outliers (S11, M11) indicates that phantom session reproducibility is not fully controlled and limits its claim as an ideal mechanical reference. In addition, the absence of Stepwise acquisitions in the Rigid phantom condition limits the completeness of the protocol comparison within the phantom arm and precludes assessment of Stepwise analytical consistency behaviour in a phantom substrate with a clearly detectable compliance transition. This comparison should be included in subsequent validation iterations. Fifth, a direct quantitative comparison between SICM Pknee and the Dorn COP in the same subjects remains to be performed. Sixth, the repetition of three consecutive insufflation trials on the same subject may introduce a progressive mechanical conditioning effect, as each trial could partially pre-stretch the abdominal wall and leave the subsequent trial performed on a marginally more distended substrate. This effect is acknowledged as a potential source of intra-session variability. Its biomechanical magnitude is, however, expected to be limited: Chik et al. [19] demonstrated in 50 dogs that deliberate abdominal wall prestretching—performed at 10 mmHg for 3 min—produced a median increase in intra-abdominal working space of only 6.9%, corresponding to approximately 7 mm per 10 cm of abdominal dimension. This represents the upper bound of a systematic and intentional prestretching manoeuvre under optimised conditions; any unintentional distension from sequential trials at lower pressures would be expected to produce smaller effects and is unlikely to substantially confound the parametric comparisons reported here. Seventh, the effective acquisition rate of 15–16 Hz is adequate for quasi-static P–V curve reconstruction under the experimental conditions tested, where the dominant biomechanical signal components evolve over seconds and are well below 1 Hz. However, the sufficiency of this sampling rate for in vivo applications has not been assessed in the present study. In live anaesthetised subjects, respiratory motion, cardiac pressure oscillations, and active insufflator regulation cycles introduce higher-frequency pressure components that may require higher acquisition rates or adaptive filtering strategies to resolve without aliasing. This aspect will need to be formally evaluated in the transition to live-subject validation, and higher sampling rates are under consideration for the next hardware iteration.

## 5. Conclusions

This pilot study demonstrates the technical feasibility of the SICM inline module for real-time acquisition and biomechanical characterisation of the abdominal P–V curve during CO_2_ pneumoperitoneum in controlled experimental models. Across 48 curves acquired in a two-arm design—a biomechanical phantom benchmark and a feline cadaveric model—the system consistently produced high-quality pressure and flow signals, with volume integration error below 0.1% relative to an independent reference. Under the Continuous insufflation protocol, key biomechanical parameters including maximum compliance (Cmax), pressure at maximum compliance (PCmax), and Knee Pressure (Pknee) were extracted with intra-session reproducibility consistent with a preliminary validation study. The Rigid phantom and cadaveric datasets showed overlapping parameter ranges, supporting the coherence of the analytical consistency across physically distinct substrates. The Stepwise protocol, while not fully supported by the current extraction analytical consistency in all subjects, provided methodologically informative boundary-condition data that directly informs the next development stage.

The present findings do not establish clinical utility, define optimal insufflation pressure targets, or demonstrate superiority over existing methods. They provide instead a structured pre-clinical foundation—across two levels of a progressive validation analytical consistency—for the development of a quantitative, patient-specific approach to abdominal mechanics monitoring in veterinary laparoscopy.

## 6. Patents

L.L. is the inventor of European Patent Application No. EP26164347.2 (application date: 12 March 2026; status: pending), titled “Method and System for Real-Time Evaluation and Visualization of Physiological and Biomechanical Parameters During the Performance of a Laparoscopic Pneumoperitoneum Operation,” filed with the European Patent Office by Università degli Studi di Bari Aldo Moro, Bari, Italy. The authors do not believe the patent or any future benefits they may receive because of it inappropriately influence any work described in this manuscript. The other authors declare no conflicts of interest.

## Figures and Tables

**Table 1:** Biomechanical parameters by stiffness and protocol—phantom arm (mean ± SD). N/R = not detectable within acquired pressure range. S11 and M11 excluded.

Rigidity	Protocol	*n*	PCmax (mmHg)	Cmax (mL/mmHg)	Pknee (mmHg)	MaxV (mL)	MaxP (mmHg)	CQI
Soft	Continuous	5	10.40 ± 0.20	714 ± 363	N/R	4077 ± 315	11.58 ± 0.15	1.0000
Soft	Stepwise	6	10.25 ± 0.25	702 ± 158	N/R	4471 ± 260	11.45 ± 0.28	1.0000
Medium	Continuous	6	10.17 ± 0.24	366 ± 66	N/R	3512 ± 332	11.47 ± 0.18	1.0000
Medium	Stepwise	5	10.50 ± 0.32	730 ± 397	N/R	4993 ± 844	11.66 ± 0.24	1.0000
Rigid	Continuous	6	2.75 ± 0.63	139 ± 18	4.08 ± 0.73	1357 ± 63	15.10 ± 0.22	1.0000

N/R: C(P) does not reach the predefined compliance drop threshold (EP26164347.2) within the acquired pressure range (~11–12 mmHg) for Soft and Medium groups. Work Zone (Rigid) = Pknee − PCmax = 1.33 ± 0.37 mmHg.

**Table 2:** Intra-group variability (CV%) by stiffness × protocol—phantom arm.

Rigidity × Protocol	*n*	CV% PCmax	CV% Cmax	CV% MaxV
Soft · Continuous	5	1.9%	50.9%	7.7%
Soft · Stepwise	6	2.4%	22.6%	5.8%
Medium · Continuous	6	2.3%	18.1%	9.4%
Medium · Stepwise	5	3.0%	54.4%	16.9%
Rigid · Continuous	6	22.9%	13.3%	4.7%

Within this phantom dataset, Stepwise yielded lower Cmax variability in the Soft condition, whereas Continuous yielded lower Cmax variability in the Medium condition. Rigid was assessed under Continuous only. These protocol-related differences are descriptive and should not be interpreted as general acquisition recommendations.

**Table 3:** Biomechanical parameters—cadaveric arm, Continuous protocol (mean ± SD, *n* = 3 technical replicates per subject). CV% reported as G1/G2/G3. BW = body weight.

Parameter	G1 (3.0 kg)	G2 (4.0 kg)	G3 (3.2 kg)	CV% (G1/G2/G3)
PCmax (mmHg)	2.00 ± 0.00	3.00 ± 0.41	2.00 ± 0.00	<1/13.6/<1
Cmax (mL/mmHg)	157.9 ± 5.8	116.8 ± 17.7	191.4 ± 13.9	3.7/15.2/7.3
Pknee (mmHg)	3.50 ± 0.00	4.17 ± 0.24	3.33 ± 0.24	<1/5.7/7.1
MaxP (mmHg)	15.97 ± 1.09	15.13 ± 0.54	15.00 ± 0.22	6.8/3.6/1.4
MaxV (mL)	886 ± 125	1070 ± 134	1137 ± 64	14.1/12.5/5.7
Cmax/BW (mL/mmHg/kg)	52.6 ± 1.9	29.2 ± 4.4	59.8 ± 4.3	3.7/15.2/7.3
MaxV/BW (mL/kg)	295 ± 42	267 ± 33	355 ± 20	14.1/12.5/5.7
Work Zone (mmHg)	1.50	1.17	1.33	—
Slope (mmHg/mL ×10^−3^)	16.7 ± 1.7	12.1 ± 2.5	13.8 ± 1.5	10.4/20.9/11.2
CQI	0.9965 ± 0.0010	0.9974 ± 0.0001	0.9963 ± 0.0010	0.1/0.0/0.1

Work Zone = Pknee − PCmax. N = 3 per subject reflects within-subject technical replicates, not independent biological observations.

**Table 6:** Cross-arm consistency check: Rigid phantom vs. feline cadaveric model—Continuous protocol. Same analytical consistency applied to both arms. Values indicate parameter overlap, not biological equivalence.

Parameter	Rigid Phantom (*n* = 6)	Feline G1 + G3 (Cont., *n* = 6)	Feline G2 (Cont., *n* = 3)	Notes
PCmax (mmHg)	2.75 ± 0.63	2.00 ± 0.00	3.00 ± 0.41	Overlapping
Cmax (mL/mmHg)	139 ± 18	175 ± 21	117 ± 18	Compatible range
Pknee (mmHg)	4.08 ± 0.73	3.42 ± 0.10	4.17 ± 0.24	Overlapping
Work Zone (mmHg)	1.33 ± 0.37	1.42 ± 0.10	1.17 ± 0.24	Compatible
MaxV (mL)	1357 ± 63	1012 ± 75	1070 ± 134	Phantom +30–34%
MaxP (mmHg)	15.10 ± 0.22	15.5 ± 1.1	15.1 ± 0.5	Overlapping
CQI	1.0000	0.9968 ± 0.0009	0.9974 ± 0.0009	Phantom superior

## Data Availability

The data supporting the reported results are available from the corresponding author upon reasonable request.

## Supplementary Materials

The following supporting information can be downloaded at: https://www.mdpi.com/article/10.3390/ani16111593/s1, Figure S1. SICM workflow.
