# Supplementary material for: Pilot Validation of a Novel Inline Device for Real-Time Monitoring of Abdominal Mechanics During Pneumoperitoneum
Source: Animals (Basel). 2026 May 23;16(11):1593. doi: 10.3390/ani16111593 (PMC13255752; doi:10.3390/ani16111593)
Supplement: Supplementary file 1 [file animals-16-01593-s001.zip › animals-4262167-supplementary.pdf]

Supplementary Figure S1

High-level SICM biomechanical parameter extraction pipeline

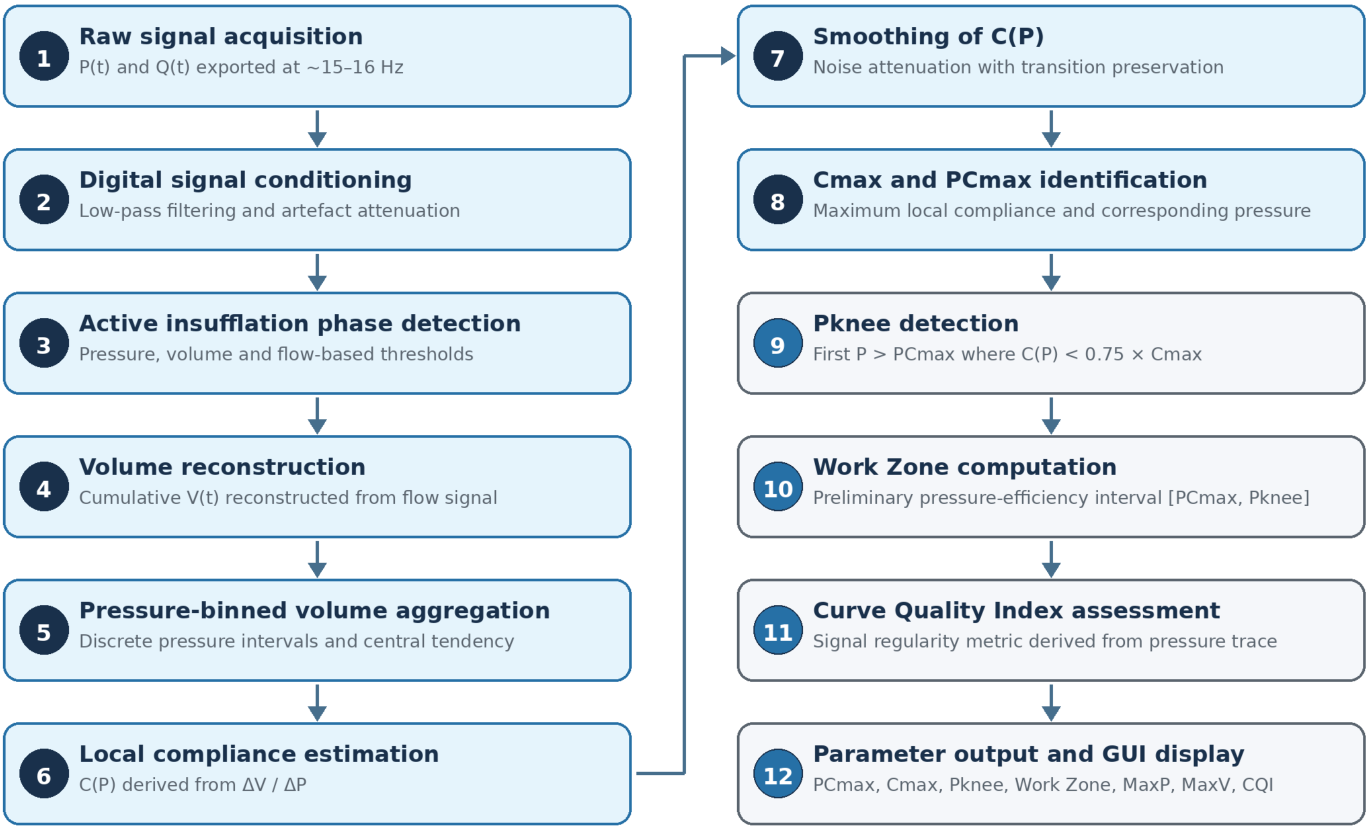

Disclosure boundary

This figure describes the logical structure of the SICM analytical pipeline. Specific implementation parameters, including signal conditioning settings, pressure-binning intervals, smoothing procedures and the CQI computation formula, are proprietary elements covered by European Patent Application No. EP26164347.2 and are not disclosed.

Caption

Supplementary Figure S1. High-level flowchart of the SICM biomechanical parameter extraction pipeline. Steps are presented in sequential order from raw pressure and flow signal acquisition to parameter output. Specific implementation parameters, including signal conditioning settings, pressure-binning intervals, smoothing procedures, and the CQI computation formula, are proprietary elements covered by European Patent Application No. EP26164347.2 and are not disclosed. The flowchart is intended to allow independent assessment of the logical structure and completeness of the analytical approach.

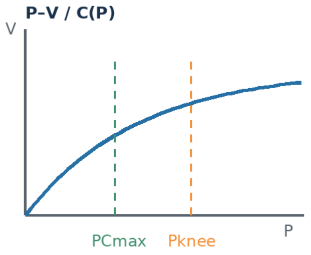

SICM = Smart Inline Compliance Module; P(t) = pressure signal; Q(t) = flow signal; V(t) = volume; C(P) = local compliance.
